# On the Origin of the Non-brittle Rachis Trait of Domesticated Einkorn Wheat

**DOI:** 10.3389/fpls.2017.02031

**Published:** 2018-01-04

**Authors:** Mohammad Pourkheirandish, Fei Dai, Shun Sakuma, Hiroyuki Kanamori, Assaf Distelfeld, George Willcox, Taihachi Kawahara, Takashi Matsumoto, Benjamin Kilian, Takao Komatsuda

**Affiliations:** ^1^National Institute of Agrobiological Sciences, Tsukuba, Japan; ^2^Plant Breeding Institute, The University of Sydney, Cobbitty, NSW, Australia; ^3^The Institute for Cereal Crops Improvement, Tel Aviv University, Tel Aviv, Israel; ^4^Centre National de la Recherche Scientifique, Saint-André-de-Cruzières, France; ^5^Plant Germplasm Institute, Graduate School of Agriculture, Kyoto University, Kyoto, Japan; ^6^Global Crop Diversity Trust, Bonn, Germany; ^7^Institute of Crop Science, National Agriculture and Food Research Organization, Tsukuba, Japan

**Keywords:** agricultural origins, einkorn, wheat, non-brittle rachis, domestication

## Abstract

Einkorn and emmer wheat together with barley were among the first cereals domesticated by humans more than 10,000 years ago, long before durum or bread wheat originated. Domesticated einkorn wheat differs from its wild progenitor in basic morphological characters such as the grain dispersal system. This study identified the *Non-brittle rachis 1* (*btr1*) and *Non-brittle rachis 2* (*btr2*) in einkorn as homologous to barley. Re-sequencing of the *Btr1* and *Btr2* in a collection of 53 lines showed that a single non-synonymous amino acid substitution (alanine to threonine) at position 119 at *btr1*, is responsible for the non-brittle rachis trait in domesticated einkorn. Tracing this haplotype variation back to wild einkorn samples provides further evidence that the einkorn progenitor came from the Northern Levant. We show that the geographical origin of domesticated haplotype coincides with the non-brittle domesticated barley haplotypes, which suggest the non-brittle rachis phenotypes of einkorn and barley were fixed in same geographic area in today’s South-east Turkey.

## Introduction

The diploid einkorn wheat *Triticum monococcum* L. subsp. *monococcum* (2*n* = 2*x* = 14, A^m^A^m^) (hereafter *Tm*) was among the first crops domesticated in the Fertile Crescent starting from its wild progenitor *Triticum monococcum* L. subsp. *boeoticum* (Boiss.) Á. Löve et D. Löve (2*n* = 2*x* = 14, A^b^A^b^) (hereafter *Tb*). The archeological record of einkorn domestication both in terms of date and place remains elusive maybe because it is a minor crop that usually is associated with emmer. However, using molecular markers, the present-day distribution of a progenitor of domestic einkorn has been located in south-eastern Turkey – between the volcanic mountain ranges of Kartal–Karadaǧ and Karacadaǧ (Heun et al., 1997; Kilian et al., 2007).

The geographical range and the long-time periods shown from the archeological evidence points to a protracted and geographically diffuse processes of domestication for most if not for all Near Eastern crops, rather than a single process (Lev-Yadun et al., 2000; Fuller et al., 2011, 2012; Willcox, 2013), however, some would disagree (Heun et al., 2012). This applies also to einkorn wheat, for which the ‘dispersed-specific model’ has been proposed (Kilian et al., 2007).

On Near Eastern archeological sites, emmer and barley were the staple cereals and those where einkorn dominate are the exception. One example is Tell Qaramel (Willcox and Herveux, 2013). Two-grained einkorn was identified at the late Pleistocene sites of Mureybet and Abu Hureyra. But there was a doubt with regard to the identification because of the confusion with wild *Secale* species. The term *Triticum/Secale* was adopted, however, we now know that this taxon is dominated by wild rye (Willcox, 2007, 2014). Einkorn on Near Eastern sites is represented predominantly by two-grained forms (Özkan et al., 2011) while on European sites the single-grained type dominates (Kreuz and Boenke, 2002). During the last millennia, hulled wheats were largely replaced by free-threshing tetraploid (durum wheat) and hexaploid wheats (bread wheat), which deliver higher yields. Today, einkorn is cultivated on a very small scale, i.e., in the Balkans, France, Italy, Morocco, Spain, and Turkey (Schiemann, 1956, 1957; Nesbitt and Samuel, 1996; Perrino et al., 1996; Zaharieva and Monneveux, 2014). This diploid wheat has been re-discovered as a source of genetic variation for wheat breeding, and it is increasingly used by the organic food industry in Europe due to its nutritional value and biodiversity that can be exploited for the improvement of the health-related traits of modern wheat (Arzani and Ashraf, 2017).

Multi-locus re-sequencing analysis of a comprehensive collection of 321 wild and 92 domesticated einkorn lines revealed, for instance, that wild einkorn consists of three distinct *Tb* races (i.e., alpha, beta, gamma) (Kilian et al., 2007). One of those races, the wild race beta, is genetically much more similar to domesticated einkorn, hence it is the presumed race that was the progenitor of all known present-day domestic einkorns. Today race beta occurs only in the Kartal–Karadaǧ and Karacadaǧ mountains. The diffusion of domesticated einkorn out of the Fertile Crescent was reconstructed based on archaeobotanical remains and genotyping data summarized by (Zaharieva and Monneveux, 2014; Brandolini et al., 2016).

**FIGURE 1:**
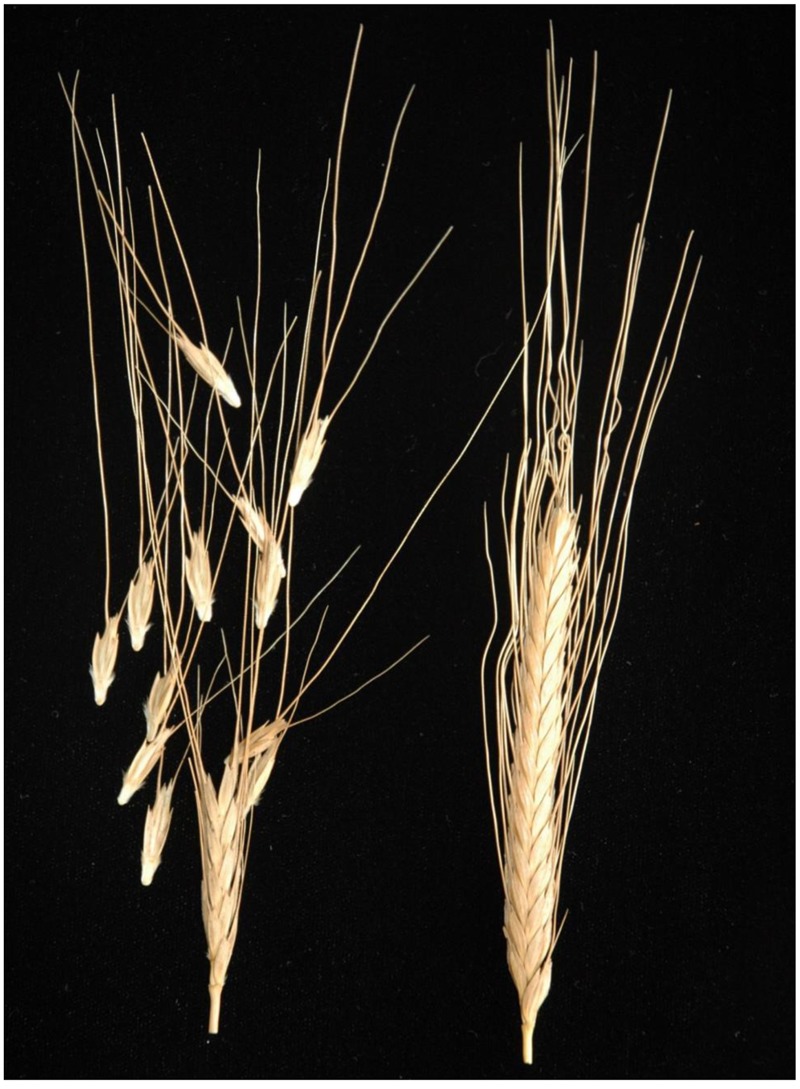
Wheat spike showing the brittle rachis of wild einkorn (*Tb*) accession KT1-1 **(Left)** and non-brittle rachis of domesticated einkorn (*Tm*) accession KT3-5 **(Right)**.

For simplicity, we follow the classification system of J. MacKey (MacKey, 1988), although wild and domesticated einkorn are not separated by any significant crossing barrier (Harlan and Zohary, 1966). The rachis type is one of the most conspicuous features distinguishing *Tb* from *Tm*: The *Tb* forms have a fragile rachis, which promotes seed dispersal in the wild, while the latter’s rachis is non-fragile and breaks when threshed (**Figure 1**), a prominent component of the domestication syndrome (Hillman and Davies, 1990). The fracture point of rachis in einkorn wheat lies above the node to produce a wedge-shape rachis node (Sakuma et al., 2011). The wedge type is found in diploid and tetraploid wheats harboring the A genome (A, AB, AG) (Li and Gill, 2006) as well as for hexaploid (ABD) (Watanabe et al., 2002). Wild einkorn wheat (*Tb*) produces wedge-shape spikelets rather similar to those of wild barley (*Hordeum vulgare* ssp. *spontaneum* (C. Koch) Thell. In barley, the brittle rachis trait is conditioned by the allelic status at two physically linked genes *Non-brittle rachis 1* (*btr1*) and *Non-brittle rachis 2* (*btr2*): a loss-of-function in either gene results in the formation of a non-brittle rachis. Loss-of-function mutations in both genes are found in the domesticated barley genepool, implying at least three independent origins of the stiff rachis phenotype in barley (Pourkheirandish et al., 2015; Civan and Brown, 2017). The objective of this study was to identify the causative polymorphism responsible for the non-brittle rachis trait of domesticated einkorn and to find closest present-day representatives of the wild ancestors of cultivated einkorn that could indicate the possible location of einkorn domestication.

## Materials and Methods

### Plant Materials

A recombinant inbred population of 115 lines (referred to as RILWA-1) bred from the cross *Tm* ‘KT3-5’ × *Tb* ‘KT1-1’ (Shindo et al., 2002) was obtained from Yokohama City University, Yokohama. Aneuploid stocks of bread wheat cv. Chinese Spring (CS) [nullisomic-tetrasomics (NTs); Sears, 1954, 1966 and ditelosomics (Dts); Sears and Sears, 1978)] were obtained from the National BioResource Project (NBRP)/KOMUGI, Kyoto University, as were a representative set of 33 accessions of *Tb* and 20 accessions of *Tm* (Supplementary Table S1). The full set of materials was fall-sown in October 2008 and 2009 in the field at Tsukuba, Japan.

### Genetic Analysis of the Non-brittle Rachis Trait

The rachis type of each of the RILWA-1 lines was characterized following the method described by Pourkheirandish et al. (2015). The awns emerging from opposing rows were pulled apart, and the proportion of brittle rachis nodes scored as 100× (the number of disarticulated rachis nodes/total number of rachis nodes). At least three mature spikes per plant of at least ten plants per line were tested. Genotypic scores for the lines, based on 211 EST markers, were obtained from Hori et al. (2007). A derived cleaved amplified polymorphic sequence (dCAPS) assay was developed to target a sequence within the *Btr1* coding region to map the *btr1* in diploid wheat: the relevant primer sequences were 5′-TCGCTCTGAGCAGGCTCGCGGCC/5′-GGTCCGGCTGTGAAGCATGAAC, and the resulting amplicons were digested with *Not*I. A linkage map (212 loci) was constructed from the genotypic data using MAPMAKER/EXP v3.0 software (Lander et al., 1987) and was used to perform a QTL analysis for rachis brittleness using Genstat v18 software^1^ (VSN International). The initial whole genome scan was performed applying a simple interval mapping (SIM) approach, which was followed up with composite interval mapping (CIM) once the appropriate co-factors had been selected.

### Sequencing and Annotation of CS Bacterial Artificial Chromosomes (BACs)

The CS whole genome shotgun sequence (Brenchley et al., 2012) was queried using the barley *Btr1* sequence (GenBank accession KR813335) in order to design a PCR assay for the wheat *Btr1* coding region. The resulting primer sequences were 5′-CCGCAACGCTGCTGGGAGTT/5′-GGAGCGCGTCCTGGGCCT. The chromosomal origin of the amplicon was explored using DNA of the set of CS aneuploid lines (NTs and Dts) as template. A bacterial artificial chromosome (BAC) library constructed from CS genomic DNA (obtained from the John Innes Centre Genome Laboratory, Norwich, United Kingdom) was screened using the PCR assay designed above and shotgun sequencing of the BACs was carried out by the standard method (International Rice Genome Sequencing Project [IRGSP], 2005). Repetitive elements (retrotransposons and DNA transposons) were discarded with the aid of Repeat Masker software^2^. Gene-specific PCR and re-sequencing primers were designed for *Btr1* and *Btr2* from the contigs of wheat cv. CS BAC clone WCS1891K06 (Supplementary Table S2).

### DNA Extraction and PCR Amplification

Genomic DNA was isolated from fresh single leaves as described elsewhere (Komatsuda et al., 1998) and used as template in a 10 μl PCR containing 0.25 U Ex*Taq* polymerase (Takara, Tokyo, Japan), 0.3 μM of each primer, 200 μM dNTP, 2.0 mM MgCl_2_, 2.5% v/v DMSO, 25 mM TAPS buffer (pH 9.3), 50 mM KCl, 1 mM 2-mercaptoethanol and 20 ng template. The PCR regime comprised an initial denaturation of 94°C/5 min, followed by 30 cycles of 94°C/30 s, 60°C/30 s, 72°C/2 min, and was completed by a final extension of 72°C/10 min.

### Amplicon Purification and Sequencing

The amplicons were purified using a QIAquick PCR purification kit (Qiagen, Germantown, MD, United States), then subjected to cycle sequencing on both strands using Big Dye Terminator v3.1 (Applied Biosystem, Foster City, CA, United States) technology. Each reaction comprised 25 cycles of 96°C/10 s, 50°C/5 s, 60°C/4 min. Salts, non-incorporated dNTPs and dye terminator were removed with an Agencourt CleanSeq system (Beckman Coulter, Fullerton, CA, United States), and the sequencing data was acquired using an ABI Prism 3130 genetic analyzer sequencer (Applied Biosystems, Foster City, CA, United States).

### Phylogenetic Analysis

Sequencing reads were imported into Sequencher v5.4 software^3^ and low quality reads were removed manually. Sequence alignments were generated using ClustalW within MEGA 6 software (Tamura et al., 2013), and haplotypes defined using DnaSP 5.10.01 software (Librado and Rozas, 2009). Singleton SNPs and haplotypes (those detected in only one accession) were confirmed by manually inspecting the sequence chromatogram file.

A wild emmer wheat (*T*. *turgidum* ssp. *dicoccoides* (Körn.) Thell. ‘Zavitan’ (Avni et al., 2017) and a wild barley accession (*Hordeum vulgare* ssp. *sponaneum*, ‘OUH602’) were used as outgroups. The resulting phylogenetic trees were constructed using MEGA 6 software (Tamura et al., 2013) based on the neighbor-joining method, with uniform rates among sites, and applying the pairwise-deletion option to deal with gaps/missing data. A bootstrap analysis (1,000 replicates) was performed to provide confidence estimates for branch nodes.

## Results

### The Genetic Basis of the Non-brittle Rachis Trait in Einkorn Wheat

**FIGURE 2:**
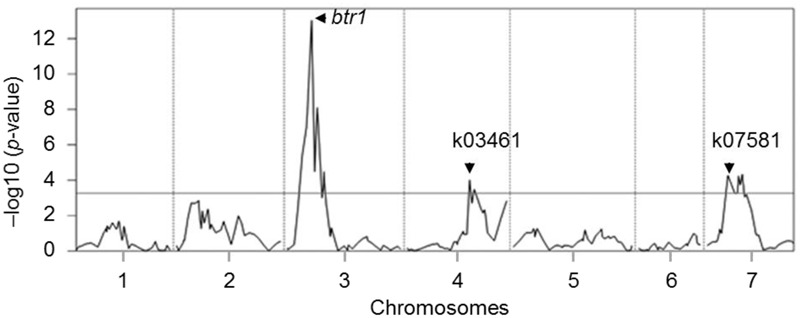
QTL mapping for rachis brittleness in einkorn wheat. The mean (across two seasons) rachis brittleness score was used to represent the trait in the analysis. The horizontal line represents the LOD threshold. The *btr1* (dCAPS), k03461, and k07581 are molecular markers linked to the QTLs.

Phenotypic evaluation over two growing seasons showed that the *Tb* accession KT1-1 formed the wedge type brittle rachis in 67.0% rachis nodes, while the *Tm* accession KT3-5 formed the wedge type brittle rachis in only 1.5% rachis nodes (**Figure 1**). In the RILWA-1 population, the trait showed a continuous frequency distribution, but can be divided equally between those falling into the range 0–20% (non-brittle lines) and 50–90% (brittle lines), consistent with major locus effect that control over the trait (Supplementary Figure S1). QTL analysis revealed that rachis brittleness was determined by three loci, mapping to chromosomes 3A, 4A, and 7A (**Figure 2** and Supplementary Table S3). The largest effect locus co-located with *btr1* (dCAPS) at position 36.7 cM on chromosome 3A; the locus was associated with a LOD of 15.2 and explained 44.7% of the variance. The sequences of the two allelic forms of BTR1 revealed an alanine in wild einkorn (KT1-1) to threonine in domesticated einkorn (KT3-5) substitution at position 119. Whereas in BTR2 revealed an aspartic acid in wild einkorn (KT1-1) to glutamic acid in domesticated einkorn (KT3-5) substitution at position 10.

### The *Btr1* and *Btr2* Homologs in Hexaploid Wheat

**FIGURE 3:**
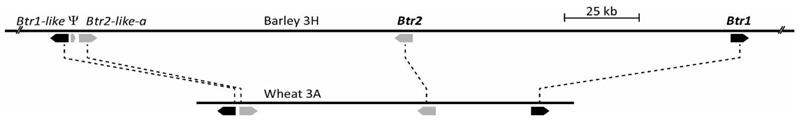
Comparison of orthologous *Btr1*/*Btr2* loci from wild barley OUH602 (**Upper**, accession KR813335, Pourkheirandish et al., 2015) and bread wheat cultivar Chinese Spring (CS) A genome (**Lower**, accession MG324346, this study). *Btr1* and *Btr1-like* are marked by black arrows, *Btr2* and *Btr2-like* by gray arrows, while Ψ is a pseudogene sharing some homology with *Btr2*.

The primer pair 5′-CCGCAACGCTGCTGGGAGTT/5′-GGAGCGCGTCCTGGGCCT amplifies a 249 bp amplicon using a template of CS genomic DNA. An aneuploid analysis showed that the only templates, which failed to amplify this fragment were those which lacked the short arm of chromosome 3A (NT3A3B, NT3A3D and DT3AL, data not shown). The 126,452 bp sequence of CS BAC clone WCS1891K06 (GenBank accession MG324346), which tested positive for this amplicon, featured four *Btr* homologs, namely *Btr1, Btr2, Btr1*-*like*, and *Btr2*-*like*. *Btr1* and *Btr2* were orientated head-to-head, separated by 37,305 bp, while *Btr1*-*like* and *Btr2*-*like* were also orientated head-to-head, but were separated by just 440 bp (**Figure 3**).

### Sequence Variation at *Btr1* and *Btr2* Genes in Einkorn Wheat

**FIGURE 4:**
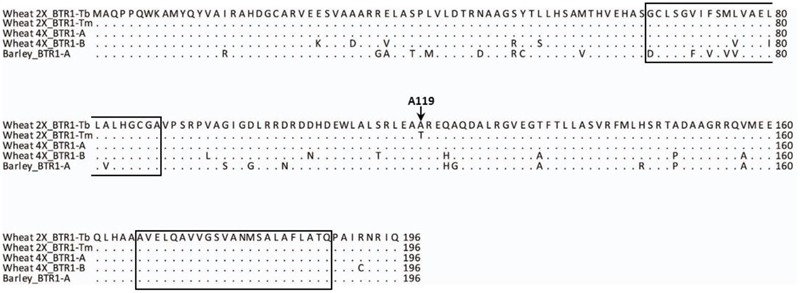
BTR1 peptide alignment of wild diploid and tetraploid wheat and wild barley. The A119 residue is conserved across the species except for *Tm*. The two predicted transmembrane helices are indicated by boxes. Wheat 2X_BTR1-Tb stands for *Tb* accession KT1-1, Wheat 2X_BTR1-Tm stands for *Tm* accession KT3-5, Wheat 4X_BTR1-A and -B *T. turgidum* ssp. *dicoccoides* ‘Zavitan’, and Barley_BTR1-A is wild barley OUH602.

The nucleotide sequence of CS (WCS1891K06) was exploited to design PCR primers suitable for re-sequencing einkorn *Btr1* (*TmB1*) and *Btr2* (*TmB2*) full genes (Supplementary Table S2). The einkorn *Btr1* sequence was found to harbor a single 591 bp exon, predicted to encode a 196-residue protein (**Figure 4**), just as its wild barley ortholog does (Pourkheirandish et al., 2015). The *Btr1* coding sequence present in KT1-1 was 91.7% identical to that present in wild barley, and the level of identity at the peptide level was 87.8%. The predicted transmembrane helices were highly conserved between barley and einkorn wheat.

The einkorn *Btr2* sequence was found to harbor a single 597 bp exon, predicted to encode a 198-residue protein, while the wild barley ortholog features a single exon of length 609 bp (202 residue product). The equivalent comparison between the KT1-1 and the wild barley *Btr2* coding sequences revealed an 88.0% level of nucleotide identity and a level of identity at the peptide level of 82.0%.

**FIGURE 5:**
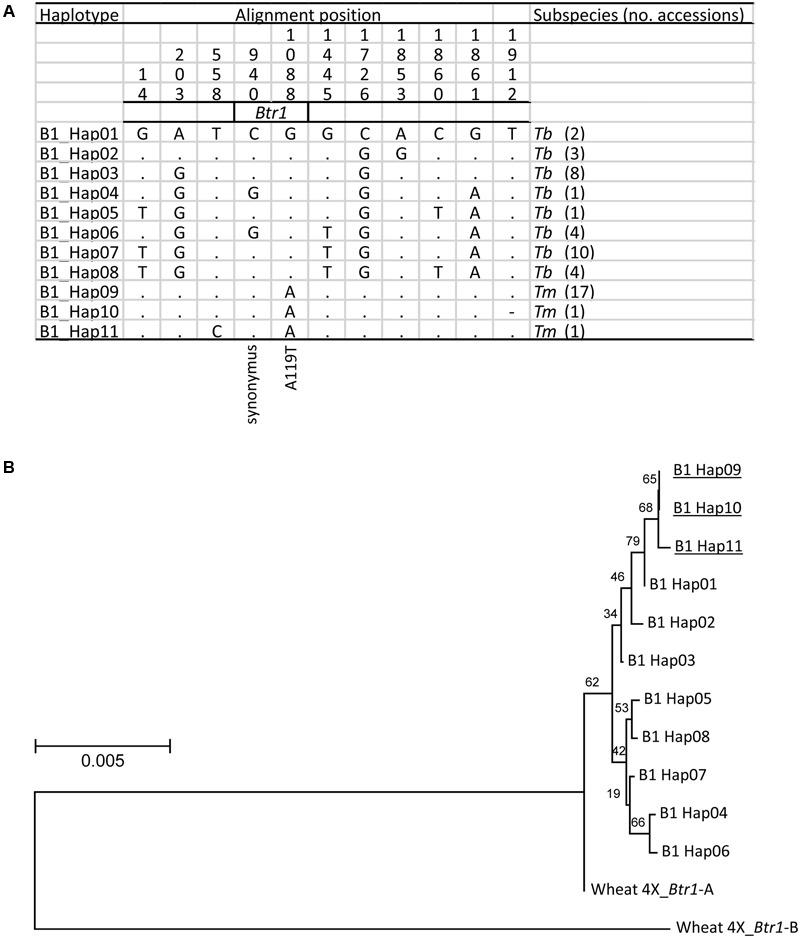
The origin of the *btr1* allele found in domesticated einkorn wheat. **(A)** The eleven recognized *Btr1* haplotypes found in *Tm* (three haplotypes)and *Tb* (eight haplotypes). Identical nucleotides are shown in the first line as dots. The A119T amino acid substitution caused non-brittle phenotype in *Tm*. **(B)** A phylogenetic analysis of *Btr1* based on nucleotide sequence spanning the *Btr1* coding sequence plus 750 bp upstream and 900 bp downstream of the coding sequence. Wheat 4X_*Btr1*-A and -B *T. turgidum* ssp. *dicoccoides* ‘Zavitan’ (A and B genome) formed the paraphyletic outgroup. Domesticated haplotypes are indicated by underline. Local bootstrap values after 1000 replicates are indicated near the branches.

When the 2,200 bp stretch spanning the einkorn *Btr1* gene which includes 750 bp upstream and 900 bp downstream of the coding sequence was re-sequenced in a set of 20 *Tm* and 33 *Tb* accessions (Supplementary Table S1); eight *Tb* and three *Tm* haplotypes were found (**Figure 5A**). Two variants were identified in the coding region (**Figure 5A**), one at position 940 (C to G; S69 synonymous change), and the other at position 1088 (G to A; A119T non-synonymous change). All of the *Tb* accessions carried G at position 1088, and all of the *Tm* accessions carried A. The *Btr1* and *Btr1-like* copies of both barley (Pourkheirandish et al., 2015) and emmer wheat (Avni et al., 2017) encoded alanine at the position indicating alanine is a well-conserved residue and threonine a mutation in the protein (**Figure 4**). The data supported the hypothesis that the A119T mutation is a functional change implying that the brittle/non-brittle rachis trait in einkorn wheat is controlled by the allelic status at *Btr1.* A phylogenetic analysis suggested a single origin for the mutation and that the latter (TmB1_Hap09, _Hap10 and _Hap11) were derived from haplotype TmB1_Hap01 (**Figure 5B**). The two accessions (KU-10873 and KU-10901) harboring TmB1_Hap01 were collected c. 50 km southeast of Maras in southeast Anatolia/Turkey close to the Kartal–Karadaǧ Mountains (Supplementary Table S1). The analysis also showed that TmB1_Hap09 was the haplotype of the founding domesticated genotype (**Figure 5B**), which subsequently became geographically highly dispersed. The further derived haplotypes TmB1_Hap10 and _Hap11 probably arose as a result of subsequent, natural mutations in the non-coding sequence and did not cause a functional change of *Btr1* (**Figure 5A**).

**FIGURE 6:**
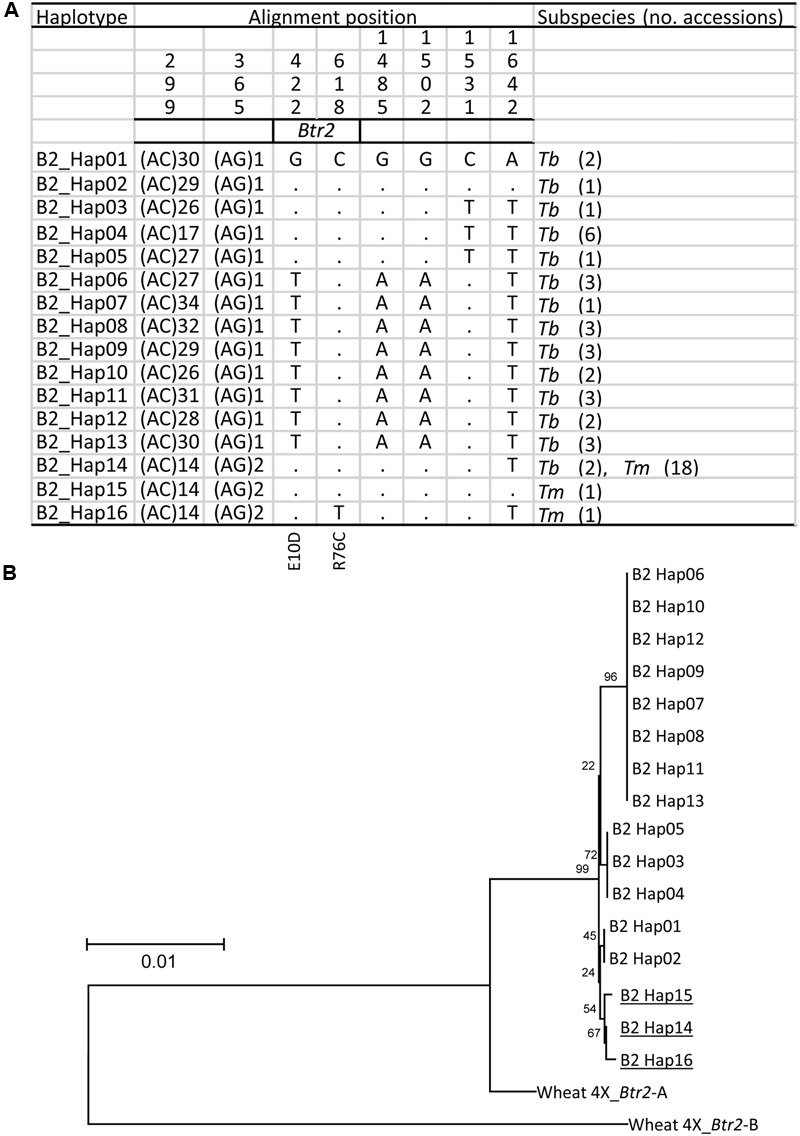
The origin of the *Btr2* allele found in domesticated einkorn wheat. **(A)** The 16distinct *Btr2* haplotypes found in *Tm* (three haplotypes) and *Tb* (14 haplotypes), of which one (B2_Hap14) is represented in both genepools. Identical nucleotides are shown in the first line as dots. **(B)** A phylogenetic analysis of *Btr2* based on nucleotide sequence spanning the *Btr2* coding sequence plus 350 bp upstream and 650 bp downstream of the coding sequence. Wheat 4X_*Btr1*-A and -B *T. turgidum* ssp. *dicoccoides* ‘Zavitan’ (A and B genome) formed the paraphyletic outgroup. Cultivated haplotypes are indicated by underline. Local bootstrap values after 1000 replicates are indicated near the branches.

Similarly, when a 1,600 bp stretch of the *Btr2* sequence which includes 350 bp upstream and 650 bp downstream of the coding sequence was re-sequenced among the *Tm* and *Tb* accessions, a total of 14 haplotypes was revealed in wild einkorn and three in domesticated einkorn wheat (**Figure 6A**); these included two non-synonymous variants in the *Btr2* coding sequence. The first of these (position 422, G to T) resulted in a predicted E10D substitution; as this polymorphism occurred within the *Tb* genepool, it cannot be responsible for the brittle rachis trait. The second non-synonymous polymorphism lay at position 618 (C to T), causing a R76C substitution. The R76C variant in *Btr2* was only found in one domesticated einkorn accession (KU-11047) originating from northern Turkey carrying haplotype B2_Hap16. It is unknown whether R76C mutation causes a functional change of *Btr2*, but this polymorphism is less likely to be responsible for the initial occurrence of non-brittle rachis in *Tm* because of its rare appearance. These findings suggest that the A119T at *Btr1* is the critical polymorphism, which causes the non-brittle rachis phenotype of domesticated einkorn.

The *Tm* haplotypes (TmB2_Hap14, _Hap15 and _Hap16) all appear to have evolved from the *Tb* haplotype TmB2_Hap14 (**Figure 6B**). As TmB2_Hap14 is the only haplotype in common between *Tb* and *Tm*, the inference is that this was the haplotype of the progenitor of the current *Tm* lineage. As expected, the two *Tb* accessions collected close to the Kartal–Karadaǧ Mountains (KU-10873 and KU-10901) were found to harbor both TmB1_Hap01 and TmB2_Hap14, a combination, which is identical to the make-up of the domesticated einkorn accessions (apart from the BTR1 A119T substitution).

## Discussion

In barley, *Btr1* and *Btr2* act as complementary genes, with loss-of-function mutations in either of them generating the non-brittle rachis phenotype (Pourkheirandish et al., 2015). In einkorn wheat, a critical A119T substitution at *Btr1* was found to discriminate between wild and domesticated einkorn wheat. A119T lies outside either of the two predicted transmembrane helices formed by BTR1, but it nevertheless appears to represent a component of the active site of the einkorn wheat BTR1 protein. The replacement of a threonine by an alanine residue has been suggested to facilitate receptor internalization in a rat opiod receptor (Wolf et al., 1999), and has also been shown to affect the activity of a histone protein (Jacob et al., 2014). Whether the A119T substitution in some way impairs the receptor-ligand structure, as suggested by Pourkheirandish et al. (2015), remains to be established. These findings suggest that the A119T at *Btr1* is the critical polymorphism, which causes the non-brittle (a stiffer) rachis phenotype of domesticated einkorn. In this study, we did not find any einkorn wheat carrying functional allele of *Btr1* in combination with loss-of-functional allele of *Btr2*. Whether *Btr1* and *Btr2* are functionally linked and act complementary genes as for barley has still to be investigated.

Today the most extensive natural stands of wild einkorn wheat are found in the northern part of the Fertile Crescent (Harlan and Zohary, 1966). However, we do not know the extent of natural stands at the end of the Pleistocene and the beginning of the Holocene. Archeological finds provide evidence for wild einkorn cultivation in northern Syria and Eastern Turkey between 11500 and 10500 cal BP. Signs of morphological domestication only appear after this long period of pre-domestication cultivation (Tanno and Willcox, 2012). Multi-locus genotyping (incl. re-sequencing) studies have concluded that the most likely site of the progenitor of einkorn wheat lies in south-eastern Turkey (Heun et al., 1997; Kilian et al., 2007). In the previous study by Kilian et al. (2007), the closest wild to domesticated einkorn were collected within the Kartal–Karadaǧ and Karacadaǧ mountain ranges on basaltic soils (Kilian et al., 2007).

Here, a haplotype analysis of *Btr1* and *Btr2* sequences showed that two wild einkorn accessions, which resembled the immediate progenitor – at *Btr1* and *Btr2* – of all domesticated einkorn wheats included in this study, both were collected between Kahramanmaraş and Gaziantep. This location lies immediately east of the Kartal–Karadaǧ mountain range and is the region where the progenitor of einkorn survives until today (Kilian et al., 2007). Furthermore, it has to be seen, if the A119T mutation led to the foundation of all known ‘eco-geographical’ or ‘geographical provenance’ groups/ lineages of domesticated einkorn (Flaksberger, 1935; Dorofeev et al., 1979; Szabó and Hammer, 1996; Zaharieva and Monneveux, 2014; Brandolini et al., 2016). Re-sequencing of *Btr1* and *Btr2* in a comprehensive collection of wild and domesticated accessions from diverse regions will answer if the non-brittle einkorn is selected only once (A119T) or multiple times.

**FIGURE 7:**
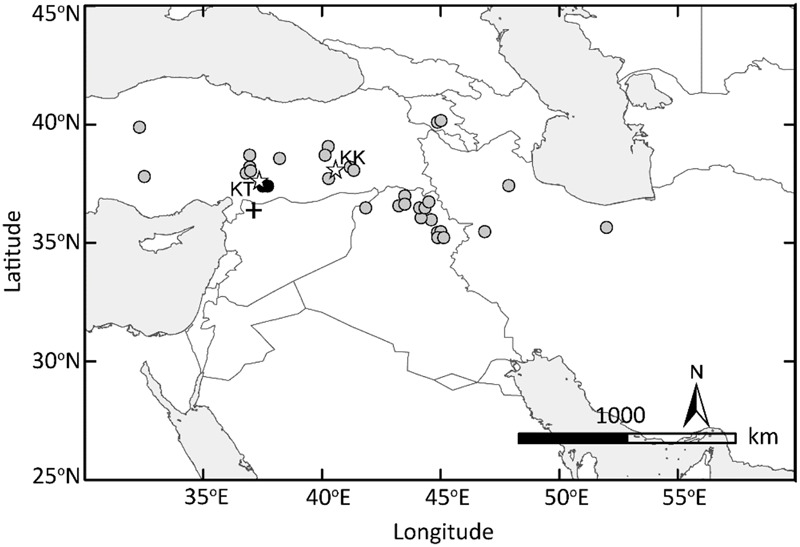
Domesticated einkorn originated in the Northern Levant. The GIS-based map of the Fertile Crescent indicates the collection sites of wild einkorn accessions analyzed in this study. Two wild einkorn lines equally closest to domesticated einkorn types at *Btr1* and *Btr2* are indicated by black dots. KT, Kartal–Karadaǧ; KK, Karacadaǧ mountains are indicated with stars. The archeological site of Tell Qaramel is indicated with plus sign.

One of the few early Neolithic sites where einkorn is the dominant crop, Tell Qaramel (Willcox and Herveux, 2013), lies less than 100 km to the south of where the two *Tb* accessions were collected (**Figure 7**). This adds weight to the hypothesis that it was in this general region that einkorn was first taken into cultivation. Willcox and Herveux (2013) suggested on the basis of an association with arable weeds that morphologically wild einkorn was cultivated at Tell Qaramel by approximately 11500 cal BP. Could this be the earliest example of cultivated wild einkorn, which later diffused as a minor component of emmer fields over a wider area of northern Levant and into Cyprus by 10600 cal BP? More archaeobotanical evidences are needed to answer this question but we can speculate that at some point the *btr1* allele appeared, fixed and dispersed to agrarian societies far away from the Karadaǧ mountain range. Einkorn is diploid and mutation in *Btr1*, rather than accumulated mutations in multiple loci or multiple genomes, was sufficient to produce non-brittle rachis. Therefore, producing non-brittle rachis seems to be achieved in rather a shorter time than domestic emmer wheat which required fixation of two non-functional mutations in the homoeologous *Btr1* genes in the A and B sub-genomes (Avni et al., 2017). The possible unintentional fixation of non-brittle rachis in diploid wheat or barley may led to an intentional selection of two mutations in tetraploid wheat genome A and B. It is true that QTLs other than the *btr1* were identified in wheat and barley but with a milder phenotypic effect (Komatsuda et al., 2004; Jiang et al., 2014).

Archaeobotanical finds indicate that it probably took over one millennium between the start of cultivation and the establishment of populations dominated by semi-brittle types (Tanno and Willcox, 2012). These finds provide evidence for multiple “false starts” and “dead ends” in the long history of plant and animal domestication, i.e., early domesticated lineages went extinct or diffused out of their original domestication area – and became slowly adapted to other environments (Fuller et al., 2011, 2012). In other words, we do not know how many domesticates were lost and we do not know to what extend the modern crop genepool (incl. the crop wild relatives) represents the situation in the past. However, Kilian et al. (2007) presented evidence that einkorn, in contrast to more intensely bred crops, possibly underwent little reduction of diversity during domestication.

The wild ancestors of domesticated einkorn *btr1* (A119T) have been found in the same geographical area as the immediate wild ancestors of the *btr2* (Pourkheirandish et al., 2015) and *btr1b* (Civan and Brown, 2017) lineages of domesticated barley. More precisely, the closest wild relatives to these non-brittle rachis phenotypes were all found northwest and southwest of Gaziantep and one of which (wild barley accession FT730) was collected in the Kartal–Karadaǧ mountains. This coincidence supports the ‘Cradle of Agriculture’ theory of Lev-Yadun et al. (2000) and in this case similar results should be found for the other founder crops of the Near East (Emmer wheat, pea, chickpea, lentil, and bitter vetch).

The genomic organization of *Btr1*/*Btr2* and *Btr1-like*/*Btr2-like* gene pairs on chromosome 3A of hexaploid bread wheat cultivar CS was exactly the same as that on barley chromosome 3H (Pourkheirandish et al., 2015). This demonstrates that the configuration must have been present in the common ancestor of wheat and barley, which diverged some eight million years ago (Middleton et al., 2014). The implied duplication event, which formed the *Btr1/Btr2* and the *Btr1-like/Btr2-like* pairs must therefore have occurred prior to the separation of the wheat and barley lineages. The sequence of the einkorn *Btr* genes is highly homologous to that of their barley orthologs. Their predicted products are of the same size and its BTR1 protein forms the same two transmembrane helices seen in both barley and emmer wheat BTR1 (Pourkheirandish et al., 2015; Avni et al., 2017). As for BTR2, neither the ‘CAR’ nor the ‘PIP’ motifs found in barley were reproduced in the wheat protein of different ploidy levels. The wild forms of both einkorn and emmer wheat, just as does that of barley produce spikelets with a wedge type rachis internode, promoting spike disarticulation above each rachis node (Zohary et al., 2013). The implication is that the products of the wild type allele at each of the *Btr* orthologs all act similarly to form a disarticulation layer above the rachis node to produce a wedge-shaped rachis internode. This form of brittle rachis is produced by species belonging to about half of the Triticeae genera (specifically, the genera *Hordeum, Triticum, Secale, Psathyrostachys, Heteranthelium, Crithopsis, Taeniatherum, Thinopyrum, Dasypyrum, Eremopyrum*, and *Sitanion*, along with a few *Aegilops* species; Sakuma et al., 2011). The extrapolation that allelic variation at the *Btr1* and/or *Btr2* orthologs is universally responsible for the brittle rachis in these taxa will need to be verified by further research.

## Conclusion

The non-brittle einkorn wheat originated by a single nucleotide change at *Btr1* that caused an amino acid substitution. How the single amino acid substitution (A119T) can change the specificity of BTR1 protein is an open question. This mutation was selected as a result of cultivation by early farmers. Today the nearest wild relatives of non-brittle einkorn have been found growing only in the northern Levant suggesting the latter may have originated there too along with non-brittle barley. The work described here provides a foundation for future studies on spike disarticulation of wheat and other members of Triticeae tribe.

## Author Contributions

TKa selected and provided the *Tb* and *Tm* materials. MP and SS assessed rachis type and performed QTL analysis. MP constructed the physical contig, which was sequenced by HK, TM, and annotated by MP. FD and MP carried out haplotype analysis. AD provided the tetraploid sequence of *btr1* and *btr2* orthologs. GW provided the archaeobotanical data. MP and TKo designed the experiments. MP, AD, BK, GW, and TKo wrote the manuscript. All authors have reviewed and commented on the manuscript.

## Conflict of Interest Statement

The authors declare that the research was conducted in the absence of any commercial or financial relationships that could be construed as a potential conflict of interest.

## Footnotes

1 http://www.vsni.co.uk/software/genstat/

2 www.repeatmasker.org/cgi-bin/WEBRepeatMasker

3 www.genecodes.com/
