# Supplementary material for: On the Origin of the Non-brittle Rachis Trait of Domesticated Einkorn Wheat
Source: Front Plant Sci. 2018 Jan 4;8:2031. doi: 10.3389/fpls.2017.02031 (PMC5758593; doi:10.3389/fpls.2017.02031)
Supplement: Supplementary file 2 [file Table_2.docx]

**Table S2.** PCR primers used for amplication and re-sequencing.
