# Supplementary material for: On the Origin of the Non-brittle Rachis Trait of Domesticated Einkorn Wheat
Source: Front Plant Sci. 2018 Jan 4;8:2031. doi: 10.3389/fpls.2017.02031 (PMC5758593; doi:10.3389/fpls.2017.02031)
Supplement: Supplementary file 3 [file Table_3.docx]

**Table S3.** Quantitative trait loci detected for rachis brittleness in RILs.
