# Supplementary material for: On the Origin of the Non-brittle Rachis Trait of Domesticated Einkorn Wheat
Source: Front Plant Sci. 2018 Jan 4;8:2031. doi: 10.3389/fpls.2017.02031 (PMC5758593; doi:10.3389/fpls.2017.02031)
Supplement: Supplementary file 4 [file Data_Sheet_1.docx]

**
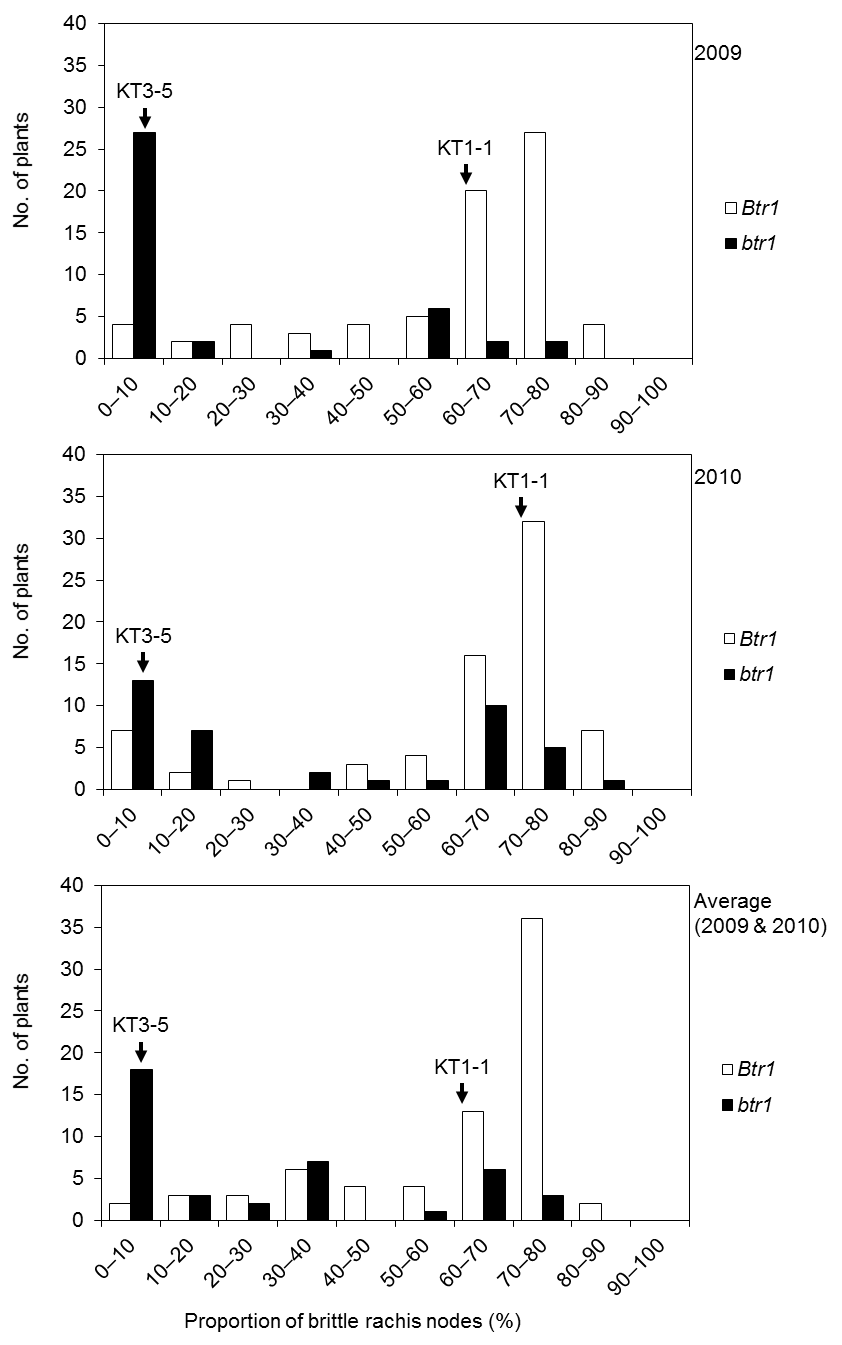
**

**Fig. S1.** Frequency distribution of brittle rachis nodes in the mapping population. The 115 F_10_ RILs derived from a cross between KT1-1 (*Tb*) and KT3-5 (*Tm*) were analyzed. Bar colors represent genotype based on the dCAPS assay of molecular marker developed for the causal point (A119T) of einkorn *Btr1;* KT1-1 (*Btr1*) and KT3-5 (*btr1*).
